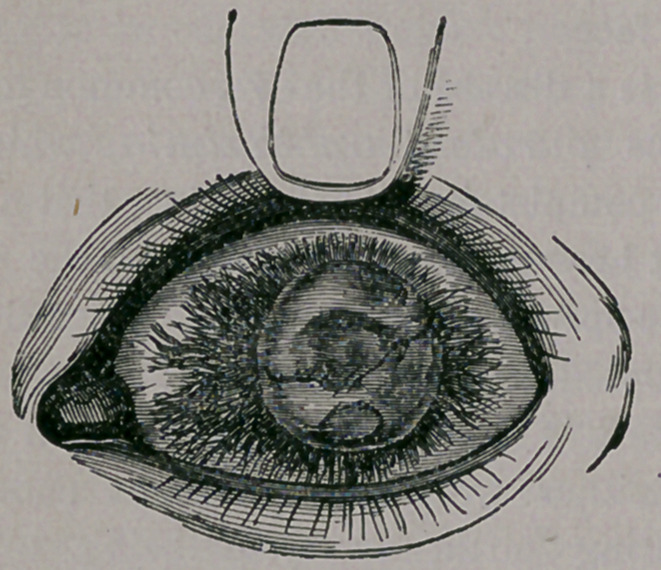# Infants' Eyes

**Published:** 1876-04

**Authors:** 


The Bistoury.

ELMIRA, N. Y., APRIL, 1876.

The BISTOURY is published Quarterly, upon the 1st of April, July, October and January, at Fifty Cents a year, in advance.

### INFANTS' EYES.

The delicate eyes of the newly born infant are naturally more susceptible to disease than those of grown folk, the only wonder is that blind babies are not more frequently encountered. Often, the child is given over to the care of inexperienced nurses, fondling and noodling grandmas or aunties, who must examine the little newcomer's eyes by the light of the window or the brilliant gas-jet, to see whether they are the color of pa's or ma's and what the particular inclination of its nose may be. All the female relatives within hailing distance, rush to its apartments, fondle it with their cold hands, and hug it to their chilled garments, until the little creature "contracts a cold," and pays the forfeit with inflamed and painful eyes. In very many instances the simple exposure of the sensitive eyes to the light of the unshaded window is sufficient to excite an inflammation that may result most disastrously to the vision.

When the young infant's eyes become irritated from "taking cold," it will tightly close them when exposed to the light, hot tears will fall over the cheeks, and the lids will appear puffed and of a purple tinge. Separate the lids with your fingers, and you will find numerous red and congested vessels running over the white portion of the eye, toward the *cornea*, as here represented:

To allow such an inflammation to progress, would be highly dangerous, as tending toward congestion, and perhaps ulceration of the delicate *cornea*. If no ophthalmic surgeon is at hand, the following collyrium may be instilled into the eye, three or four times daily, from the point of a camel's hair brush: Take rose water, one ounce; pulverized borax, three grains; sulphate of atrophia, one grain; place the ingredients in a perfectly clean bottle, and use as directed. Pledgets of lint may be soaked in cold cream, and laid over the eye-lids, and frequently changed. The infant should be kept in a well ventilated and shaded room. No bright light should be allowed to fall upon its face.

There is a disease of the eye, common to infants, known as *purulent ophthalmia*, which is so quick to completely ruin the vision, that a description of it here becomes necessary, to the end that it may be speedily recognized, and suitable treatment adopted in the early stages of the attack. This disease is induced by contamination from the mother, either from the secretions entering the eyes during birth, or from being introduced through sponges, cloths, or vessels that were used in bathing the mother. Too much care cannot be exercised with regard to this matter. It is imperatively necessary that the infant should have its own sponge, basins or other [illegible]rticles used in its bathing. The nurse should always thoroughly wash her hands with soap, after having bathed the mother, before she pays her attention to the infant. In this manner, contagion can be prevented and the risks from this terrible disease averted. When the inoculation has occurred, however, the greatest care must be used to keep it from other members of the family, and from the remaining eye of the child, where the disease is confined to one, as is usually the case. In the beginning of the disease, the eye-lids appear puffed, red and inflamed at their margins. The child keeps the eyes tightly closed, particularly when exposed to the light. In twenty-four hours after noting these symptoms, matter begins to flow from the eyes, first of a flaky nature and white of color. During the succeeding twenty-four hours the lids become greatly swollen, with an immense discharge of thick, yellowish matter, constantly gushing from between them. If it be allowed to rest upon the cheek, it will excoriate the skin and render sore every spot it touches. Later, the nostrils become sore, the nose discharges the same sort of matter, while the lids become still more swollen and more inflamed about their margins. Three days duration of the disease in this stage, will utterly ruin the eyes, and consign the innocent little one to utter and incurable blindness. If it is possible to separate the lids, in order to view the cornea, it will be found to present a highly vascular appearance, with its clearness entirely obliterated, and instead a soft, irregular mass of matter, similar to what is below shown.

As this terrible disease is constantly occurring in all parts of the country, often in places too far remote from ophthalmic surgeons, or intelligent general physicians, dooming their helpless little ones to blindness for lack of proper care, we subjoin a simple but effective treatment, which will arrest the disease if early applied.

First, the diseased eye must be thoroughly cleansed from the matter. This is best accomplished, by using equal parts of warm water and milk, which may be injected between the lids with a small syringe. Then procure a collyrium composed of rose-water, one ounce; sulphate of atropia and sulphate of zinc, of each, two grains. Dissolve the sulphates in the rose water, and apply to the eye, with a camel's hair brush, every hour, first having removed all the matter in the manner described. Next, procure another solution, containing pulverized borax, one-half ounce; soft water, (in which flax-seed has been allowed to remain over night), one pint. To this add twenty drops of Calvert's solution of carbolic acid. Shake the mixture thoroughly, saturate pledgets of lint with it, and apply them to the lids, constantly. If but one eye is affected—which is usually the case in the beginning of the disease—the sound one should be securely sealed with a compress of wet lint, over which a flannel bandage may be placed. No cloths, water, or other materials used about the diseased eye should be allowed to come in contact with the sound one. The pledgets of lint, as soon as removed from the eye, should be cast into the fire. The infant must have its own napkins, sponges, basin, and everything used about its eyes or person zealously guarded, that no other member of the family may become inoculated. No one with sores or scratches upon the fingers should bathe the baby's eyes, as we have seen the contagion communicated in this manner, resulting in alarming symptoms of swollen arms, chills, vomiting and coma, which, but for prompt remedial agents might have resulted disastrously. Of course, no safe treatment can be recommended for all cases. The plan we have laid down, will likely arrest the difficulty, if early applied. But should the symptoms grow worse, the swelling of the lids and the discharge of matter increase, no time should be lost in securing the advice of the nearest oculist; for, as before stated, three days, and often much less time, is sufficient to entirely destroy the eyes with a disease of this character.

## Figures and Tables

**Figure f1:**
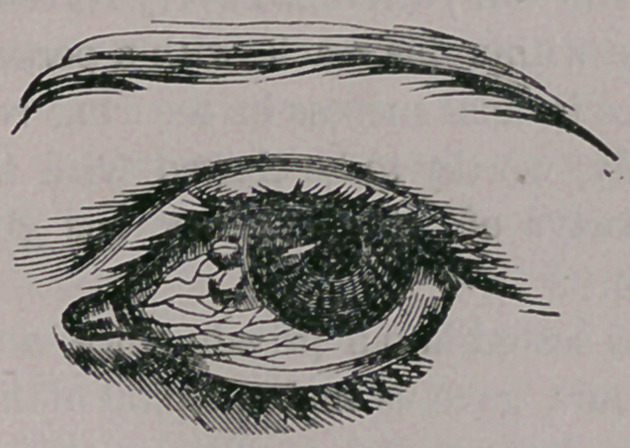


**Figure f2:**